# Sensory and motor neuronopathy in a patient with the A382P TDP-43 mutation

**DOI:** 10.1186/1750-1172-6-4

**Published:** 2011-02-05

**Authors:** Jean-Philippe Camdessanché, Véronique V Belzil, Guillemette Jousserand, Guy A Rouleau, Christelle Créac'h, Philippe Convers, Jean-Christophe Antoine

**Affiliations:** 1 Service de Neurologie, CHU de Saint-Etienne, Saint-Etienne, France; 2 Université de Lyon, F-42023, Saint-Etienne, France; 3 Centre of Excellence in Neuromics of Université de Montréal, CHUM Research Center, Montreal, Quebec, H2L 4M1, Canada; 4 Université de Montreal, Faculty of Medicine, Department of Medicine, Montreal, Quebec, H3C 3J7, Canada; 5 Research Center, CHU Sainte-Justine, Université de Montreal, Montreal, Quebec, H3T 1C5, Canada

## Abstract

Patients with *TARDBP *mutations have so far been classified as ALS, sometimes with frontal lobe dysfunction. A 66-year-old patient progressively developed a severe sensory disorder, followed by a motor disorder, which evolved over nine years. Symptoms started in the left hand and slowly involved the four limbs. Investigations were consistent with a mixed sensory and motor neuronopathy. A heterozygous change from an alanine to a proline at amino acid 382 was identified in exon 6 of the *TARDPB *gene (p.A382P). This case expands the phenotypic spectrum associated with mutations in the *TARDBP *gene and shows that sensory neurons can be severely damaged early in the course of the disease, following a propagating process, with an orderly progression from a focal starting point. A combination of severe sensory and motor neuronopathy is rarely encountered in clinical practice. The possibility of an A382P TDP-43 mutation should be considered in patients with such an association.

## Background

TDP-43, a protein involved in RNA processing, is thought to play a determinant role in the pathophysiology of amyotrophic lateral sclerosis (ALS), in which it is abnormally processed and sequestered in the cytoplasm, the perikaryon, and dystrophic neurites of neurons [1,2]. Variations in the *TARDPB *gene, which encodes TDP-43, occur in about 3% of sporadic and familial cases of ALS [3] and are mostly found in cases with a usually predominant lower motor neuron involvement and in a few patients with cognitive deficits [4-6]. We here report the case of a patient with the A382P TDP-43 mutation who developed a unique phenotype consisting of a slowly spreading sensory and motor neuronopathy.

## Case presentation

A 72-year-old right-handed non-smoker man was referred in March 2001 after a 6-year progression of a sensory, then motor, deficit in the left arm. He reported that his mother died when she was 56 after a 4-year evolution of a neurological disease that started in the upper limbs and progressively paralyzed her, including speech and deglutition. Symptoms first appeared in the patient in April 1995, with paresthesia in the left hand (month 1 = M1). In July (M4), motor deficit and muscle atrophy were absent, but 128 Hz tuning fork perception was reduced in the left index finger. Motor conduction velocities (MCV) and compound motor action potentials (CMAP) were normal in both the median and ulnar nerve, while sensory action potentials (SAP) were reduced in the left median and ulnar nerves and slightly reduced in the right sural nerve (see Additional file 1 for the electrophysiological methods and the detailed results of the ENMG study). Needle examination showed only an increased number of polyphasic motor unit potentials (MUPs) in the left abductor pollicis brevis (28% of MUPs). The cervical spine and plexus MRI was normal. Because of persistent paresthesia, somatosensory evoked potentials (SEPs) were recorded in October 1996 (M18) and were normal in the right median nerve, whereas the left N9 potential amplitude was reduced and the N13, P14, and N30 potentials abolished (Table 1).

**Table 1:** Evolution of sensory evoked potentials (SEPs) and sensory action potentials (SAPs) with disease course.

	RIGHT				LEFT			
**Months**	**4-18**	**40**	**68-72**	**104**	**4-18**	**40**	**68-72**	**104**

Median nerve								
SEPs								
N9	+	+		-	+	-		-
N13	+	-	ND	-	-	-	ND	-
P14	+	+		-	-	-		-
N20	+	+		+	-	-		-
SAP (μV) N > 8 μV	13.8	12.7	4.75	0	6.8	5	0	0

Tibial nerve								
SEPs								
N20	ND	ND	-	-	ND	ND	-	-
P30			-	-			-	-

Sural nerve								
SAP (μV) N > 8 μV	7.8	ND	6.4	6.1	8	ND	6.4	3.8

SEPs were recorded after stimulation of the median nerve at the wrist or the tibial nerve at the ankle, and SAPs were recorded in the median or sural nerves. N9, Erb point; N13, cervical spinal cord posterior horn; P14, gracilis and cuneatus nuclei; N20, parietal cortex, and P30, frontal cortex. '+' indicates a recorded potential and '-'an absent potential. ND: not done. SAPs were dromically registered with surface electrode in the ulnar nerve (head level, normal ≥ 8 μV) and antidromically in the sural nerve (foot level, normal ≥ 8 μV)

Four years later, in January 1998 (M35), motor deficit and muscle atrophy appeared in the left arm, while the sensory symptoms extended to the elbow. In July (M40), SAPs were reduced in both the median and ulnar nerve, predominantly on the left. MCVs and CMAPs were normal. Needle examination showed chronic neurogenic changes in the abductor policis brevis, abductor digiti minimi, first dorsal interosseus, and extensor carpi radialis longus in both hands, but predominantly on the left side.

In November 2000 (M68), the left arm showed diffuse muscular atrophy. Tendon reflexes were abolished and fasciculations were observed in the left biceps brachii and pectoralis major. In March 2001 (M72), the motor deficit was restricted to the left arm, with an MRC score of 3 in the deltoideus, biceps brachii, triceps brachii, and abductor pollicis brevis muscles. Muscle atrophy was restricted to the left upper limb and fasciculations were observed in all four limbs. Tendon reflexes were absent in the left arm and normal in the other limbs. The plantar response was indifferent on both sides. Bulbar involvement was absent. Tuning fork 128 Hz perception was absent at the left wrist and elbow, present at the clavicle, and reduced at the left ankle and knee. Stereognosia and graphesthesia were abolished in the left hand, while pain and thermal sensation were almost normal. There was no cognitive impairment and no dysautonomic manifestation. SAPs were absent in the left radial, ulnar, and median nerves and reduced in the other limbs. CMAPs were reduced in the left median and ulnar nerves and normal on the right side and in both peroneal and tibial nerves. Motor conduction velocities and F-wave latencies were normal. Fibrillation potentials were recorded in the upper limbs and fasciculations were present in the left vastus lateralis. SEPs were not recordable in the left median and both tibial nerves, but were normal in the right median nerve with the exception of a reduced N9 potential (Table 1).

**Figure 1:**
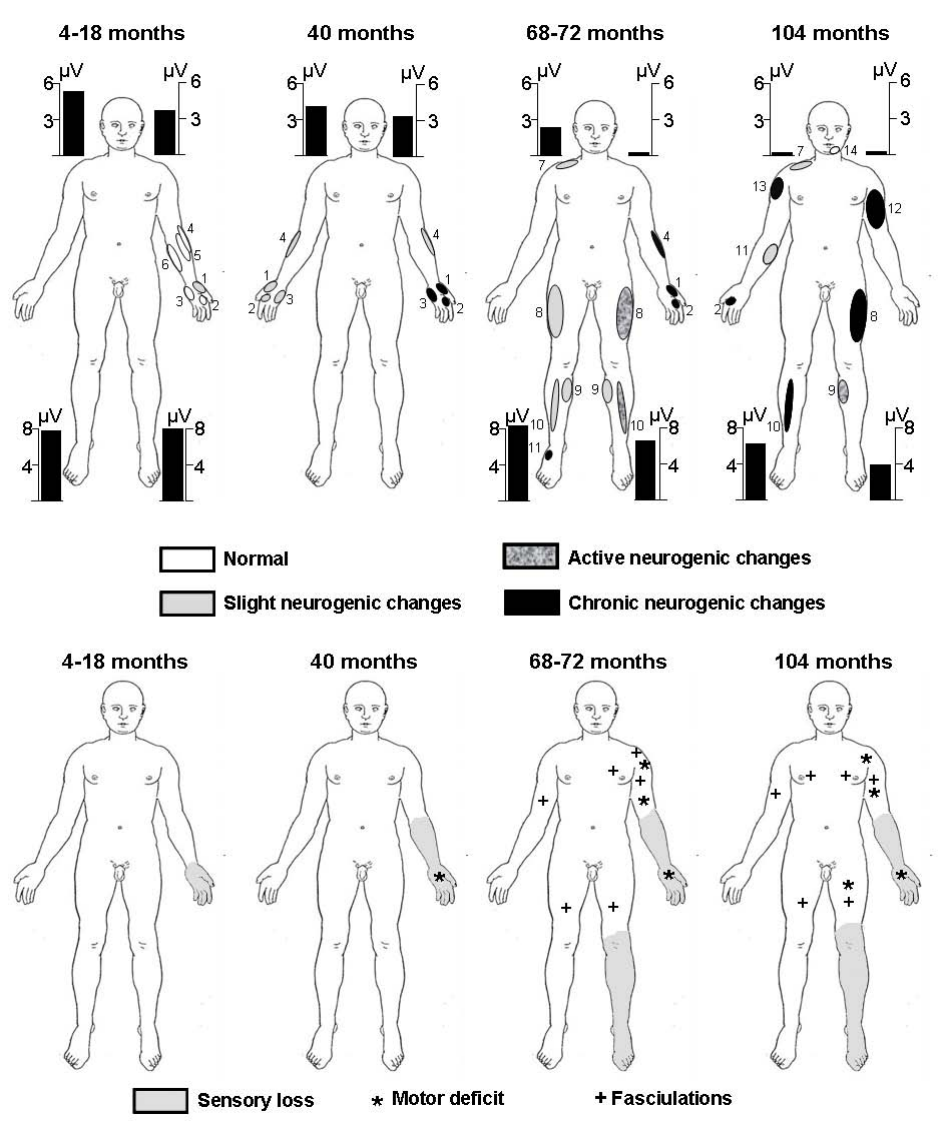
**Parallel evolution of electroneuromyographic abnormalities (top row) and clinical sensory and motor perturbations (bottom row)**. The slight neurogenic changes were motor unit potentials (MUPs) with increased polyphasism, size, and duration according to multi-MUP computer-assisted analysis, the active neurogenic changes were fasciculation potentials, fibrillations potentials, or positive sharp waves, while the chronic neurogenic changes were loss of MUPs or high firing rate. 1, abductor policis brevis; 2, first dorsal interosseus; 3, abductor digiti minimi; 4, extensor carpi radialis longus; 5, brachioradialis; 6, palmaris longus; 7, trapezius; 8, vastus lateralis; 9, gastrocnemius; 10, tibialis anterior; 11, extensor digitorum brevis; 12, biceps brachialis; 13, deltoidus; 14, mentalis. The bars correspond to sensory action potentials in μV registered dromically with a surface electrode in the ulnar nerve (head level, normal ≥ 8 μV) and antidromically with the electrode in the sural nerve (foot level, normal ≥ 8 μV).

The motor deficit slowly progressed in the left arm and, in January 2004 (M104), proximal weakness appeared in the left leg. The Norris limb score was 45/63 and the bulbar score 42/42. Forced vital capacity was normal. SAPs could not be recorded in the upper limbs and were severely reduced in the lower limbs, especially on the left side. CMAPs were reduced or absent in the left median, ulnar, tibial, and peroneal nerves and reduced in the right median and peroneal nerves. Conduction velocities were normal. Positive sharp waves and fibrillation potentials were recorded in all four limbs. SEPs were abolished in the four limbs, except in the right median nerve, where a N20 potential was recorded (Table 1). CO2 laser-evoked potentials (LEPs), evaluating Aδ and C fibers, were abolished in both hands and feet. The patient died suddenly in June 2004 (M110). No autopsy was performed. The clinical and neurophysiological data are summarized in Figure 1.

The following investigations were performed in October 2001 (M79) and were normal: cerebrospinal fluid analysis, blood cell count, ionogram, serum creatinine, glycemia, liver enzymes, vitamin E, B12 and folates, TSH, hexosaminidase A and B, amino acid chromatography, and Apo A and Apo B. Serology was negative for HIV, VDRL-TPHA, and hepatitis B and C. Screening for monoclonal gammopathy, red blood cell acanthocytosis, and antinuclear, anti-SSA, anti-SSB, anti-mitochondrial, anti-gliadin, anti-disialosyl ganglioside, and anti-onconeural antibodies, including Hu and CRMP5/CV2 antibodies, was negative. The salivary gland biopsy was normal. No mutations of the frataxin and TTR genes were found. X-ray and CT thoracic scans were normal, as was abdominal ultrasound. A muscle biopsy (left vastus lateralis) showed neurogenic changes without mitochondrial abnormalities.

**Figure 2:**
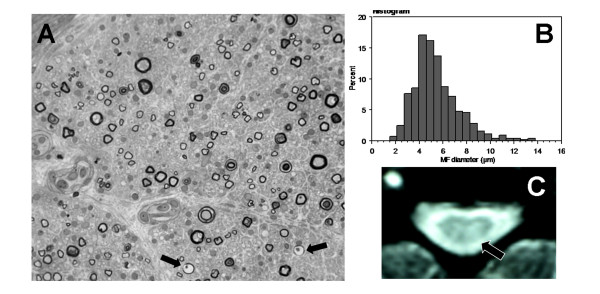
**Fiber loss mainly involved large fibers with a few degenerating axons (arrows) and no regenerating cluster as seen in the photograph of the superficial radial nerve biopsy (semithin section × 40) in (A) and the histogram in (B), (C), Abnormal hyperintensity in the left posterior column on the cervical spinal cord T2-MRI**.

**Figure 3:**
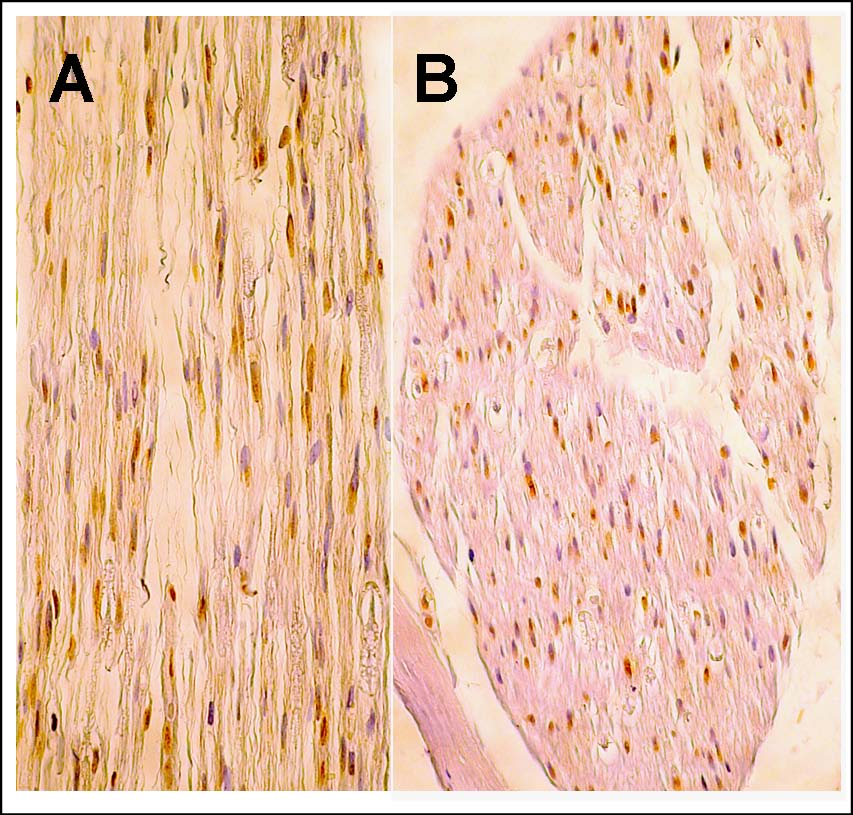
**Immunohistochemical study using anti-TDP-43 antibodies of the patient's nerve biopsy (A × 40) and a control nerve biopsy (B × 30)**. In both nerves, only Schwann cell nuclei are immunolabeled.

In December 2001 (M81), a left superficial radial nerve biopsy was normal on paraffin-embedded H-S and Congo red-stained sections. Semithin sections disclosed a severe reduction in large myelinated fiber density (Figure 2A and 2B). Only a few fibers were degenerating. Regenerating clusters were absent. Immunohistochemistry was performed on formalin-fixed paraffin-embedded samples of the nerve biopsy from the patient and, as control, a patient with a sensory neuropathy using rabbit polyclonal anti-human TDP43 antibodies (Protein Tech Group, Inc; Chicago, IL, USA) at a 1/1000 dilution. Nerve sections were deparaffinised, subjected to immunochemical detection with the antibodies, processed with a *ultra*View Universal DAB Detection Kit (Ventana Medical Systems, S.A., Illkirch, France), and counterstained with H-E. In both the patient and control, Schwann cell nuclei were immunolabeled, while no labeling was observed in the cytoplasm or in axons (Figure 3). In June 2003, the brain MRI was normal, while the T2 weighted cervical spine MRI showed a hypersignal in the left posterior column (Figure 2C).

## Genetic analysis

**Figure 4:**
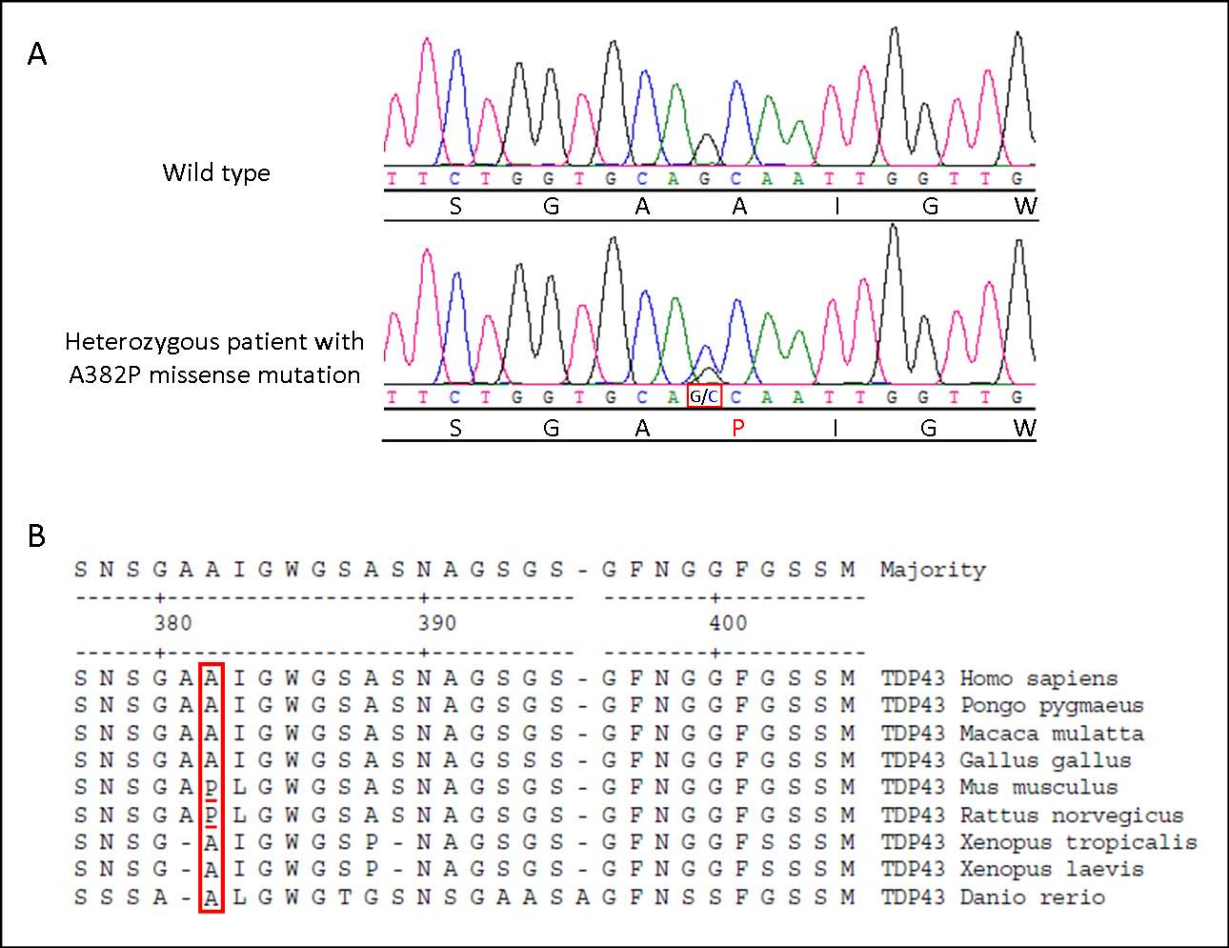
**Sequence traces and across-species conservation**. (A) Sequence trace for the p.A382P variant; the patient is shown below the wild type sequence. The red square marks the substituted nucleotide and the substituted amino acid is in red. (B) Conservation across nine species of amino acids 377 to 405 of TDP-43 is shown using the Clustal W method. Amino acid 382 is boxed in red and the proline present in the mouse and rat is underlined in red.

The sample was sequenced for mutations in the *SOD1*, *TARDBP*, and *FUS *genes. A previously reported heterozygous change from an alanine to a proline at amino acid 382 [7], resulting from the substitution of a guanine by a cytosine at position one of the codon (c.1278G > C), was identified in exon 6 of the *TARDPB *gene (p.A382P) (Figure 4). This variant was not identified in 360 control participants [8]. Family members were not available for genetic analysis. The mutated alanine is conserved in seven out of nine tested species, but is a proline in the mouse and rat (Figure 4B). Two bioinformatics programs, Polyphen [9] and SIFT [10], were used to predict the effect of the polymorphism on the protein. Polyphen predicted the change to be benign, while SIFT predicted that it would affect protein function, with a low confidence in the prediction.

## Discussion

This is the first time, to our knowledge, that a variant in *TARDBP *has been identified in a patient with a sensory and motor phenotype. It is very unlikely that this resulted from a coincidental association of sensory neuropathy and motor neuron disease, since the motor and sensory manifestations started in the same metameric territory and followed a parallel course. Moreover, an extensive work-up found no other cause of sensory neuropathy. Despite the absence of pathological evidence, the severe sensory involvement is very suggestive of sensory neuronopathy, i.e. of sensory neuron degeneration in dorsal root ganglia [11]. Thus, the sensory perturbations had a typical non-length-dependent pattern both clinically and on sensory nerve conduction study. The presence of SEPs with early absent N13 potentials and still recordable N9 potentials also suggests sensory neuronopathy [12]. Moreover, this indicates that the central process of the sensory neurons was involved before the peripheral process. Central process degeneration was also demonstrated by the presence of abnormal signals in the posterior column on spinal cord MRI, as reported previously [13]. Finally, the CO2 laser-evoked potentials indicated that, despite a predominant large neuron involvement, small nociceptive neurons were also involved.

The patient's mother's history was highly suggestive of neuromuscular disease, arguing for a hereditary transmission of the disorder. The A382P mutation identified in the patient has been reported in another ALS case, but the sensory phenotype of this patient was not specified in the publication [7]. The mutation, which was absent in 720 control chromosomes [8], was predicted to have mixed effects by two different bioinformatics software programs. The missense mutation, which is also a proline in the rat and mouse, was located in the C-terminal glycine-rich region, where most *TARDPB *mutations have been found [14]. Thus, with the available information, it can be hypothesized that the *TARDPB *variant actually caused the particular phenotype of this patient.

Sensory neuron involvement had not previously been reported for TDP-43 mutations and, when mentioned, SAPs have been reported as normal [15,16]. Pathological studies in the few available cases [4,17] and in transgenic models [18,19] did not specifically examine dorsal root ganglia, although TDP-43 is normally expressed in sensory neuron nuclei [20]. On the patient's nerve biopsy, no abnormal accumulation of TDP-43 was found in peripheral axons, in keeping with the fact that, in the central nervous system of patients with *TARDBP *mutations or sporadic ALS, the protein does not accumulate in axons, but in the neuron cell body and dendrites [1,4].

ALS is usually considered as a propagating process that recruits locally and spreads with an orderly progression [21]. In this patient, the electrophysiological results suggested a pre-existing widespread sensory dysfunction upon which the degenerative process developed focally in the left arm, then extended to the contralateral side and, through descending pathways, to the lumbar region. At each level, sensory neurons were electrophysiologically involved before motor neurons.

The combination of severe sensory and motor neuronopathy is rarely encountered in clinical practice. It has been reported in patients with paraneoplastic Hu-antibodies, [22] in facial onset sensory and motor neuronopathy (FOSMN) [23], and in rare genetic diseases, such as Tangier disease [24]. The A382P TDP-43 mutation should be considered in patients with such an association.

## Conclusions

This case broadens the spectrum of neurological syndromes associated with *TARDBP *mutations. It shows that, in rare circumstances, sensory neurons can be involved early and as severely as motor neurons and provides arguments in favor of the hypothesis that the disease followed a propagating process, with a spreading and orderly progression from a focal starting point.

## Consent

The patient gave informed consent for the genetic analysis. A copy of the written consent is available for review by the Editor-in-Chief of this journal. Consent for publication could not be obtained from the family of the deceased patient, as they could not be reached.

## Competing interests

The authors declare that they have no competing interests.

## Authors' contributions

JPC and GJ performed the electroneuromyographical investigations. PC performed the somatosensory evoked potential analysis. CC was responsible for the CO2 laser potential study. JCA performed the pathological study. VVB and GAR carried out the molecular genetic studies. JPC and JCA conceived the study, and VVB participated in its coordination and helped to draft the manuscript. All authors read and approved the final manuscript.

## Supplementary Material

Additional file 1 **Sensory and motor neuronopathy in a patient with the A382P TDP-43 mutation**. This file contains the detailed electrophysiological methods used for this study and the complete results of the ENMG study. Click here for file
