# Supplementary material for: Sensory and motor neuronopathy in a patient with the A382P TDP-43 mutation
Source: Orphanet J Rare Dis. 2011 Feb 5;6:4. doi: 10.1186/1750-1172-6-4 (PMC3042904; doi:10.1186/1750-1172-6-4)
Supplement: Additional file 1 — Sensory and motor neuronopathy in a patient with the A382P TDP-43 mutation. This file contains the detailed electrophysiological methods used for this study and the complete results of the ENMG study. [file 1750-1172-6-4-S1.DOC]

**Additional file 1**

**Sensory and motor neuronopathy in a patient with the A382P TDP-43 mutation.**

**Electrophysiological methods and results of the ENMG study.**

Jean-Philippe Camdessanché, Véronique V. Belzil, Guillemette Jousserand, Guy A. Rouleau, Christelle Créac’h, Philippe Convers, Jean-Christophe Antoine.

**Methods:**

***Electroneuromyography***

It was performed 4, 40, 68, 72 and 94 months after the onset of neurological symptoms as reported by the patient. Motor distal latencies, compound motor action potential (CMAP), motor conduction velocities and minimal F wave latencies were recorded with classical procedure in median and ulnar nerves in the forearms and, peroneal and tibial nerves in the legs. Sensory action potentials (SAP) were recorded with an antidromic procedure for median, ulnar, radial, superficial peroneal and sural nerves. Needle examination was performed with a concentric needle electrode. Muscles were selected for recording according to the topography and evolution of the motor deficit.

***Somatosensory evoked potentials (SEPs)***

They were recorded 18, 68, and 84 months after the onset neurological symptoms as reported by the patient. SEPs were recorded after stimulation of the median nerve at the wrist or tibial nerve at ankle following published recommendations [1]. For median nerves, the following components were recorded using different channels: N9 (Erb point), N13, (posterior horn of the cervical spinal cord); P14 (Gracilis and Cuneatus nuclei), N20 (parietal cortex) and P30, (frontal cortex). For tibial nerves, the N20 (parietal cortex) and P30, (frontal cortex) were recorded.

***Painful CO2 laser potentials.***

They were recorded 84 months after the onset neurological symptoms as reported by the patient. Cutaneous heat stimuli were delivered by a CO2 laser (10.6 mm wavelength, 25 W maximal output power, 3.5 mm beam diameter). These stimuli were applied to the dorsum of the hand, in the radial nerves territory and on the dorsum of the foot in the peroneal nerve territory. Two runs of 30 stimuli were performed. The inter-stimulus interval varied randomly between 4500 and 5500 ms. The stimulus duration was set at the subjective pain threshold level and was kept constant throughout the whole recording session. The laser beam was slightly moved between two successive stimuli to avoid habituation. The analysis time was 512 ms; the signal was bandpass filtered between 1 and 250 Hz and sampled at 500 Hz.

**Results**

**Electroneuromyography**

| **Sensory action potentials (µV)** | | |  |  |  |  |  |  |
| --- | --- | --- | --- | --- | --- | --- | --- | --- |
| **Months** | **L median**  **N 8 µV** | **L ulnar**  **N 8 µV** | **L radial**  **N 18 µV** | **R median**  **N 8 µV** | **R ulnar**  **N 8 µV** | **R radial**  **N 18 µV** | **L sural**  **N 8 µV** | **R sural**  **N 8 µV** |
| 4 | 6.8 | 4.2 | 5.1 | 13.8 | 5.3 | 14.8 | 8 | 7.8 |
| 40 | 5 | 3.79 | ND | 12.7 | 4.54 | ND | ND | ND |
| 68 | 0 | 0 | 0 | 4.75 | 2.53 | 5.22 | 6.39 | 6.39 |
| 72 | ND | ND | ND | ND | ND | ND | 4.57 | 8.16 |
| 94 | 0 | 0 | 0 | 0 | 0 | 3.4 | 3.8 | 6.1 |
|  |  |  |  |  |  |  |  |  |
| **Compound motor action potentials (mV) / Motor conduction velocities (ms^-1^)** | | | | | |  |  |  |
| **Months** | **L median**  **N 4mv/45 m/S** | **L ulnar**  **N 4mv/45 m/S** | **R median**  **N 4mv/45 m/S** | **R ulnar**  **N 4mv/45 m/S** | **L tibial**  **N 4mv/40 m/S** | **L peroneal**  **N 2mv/40 m/S** | **R tibial**  **N 4mv/40 m/S** | **R peroneal**  **N 2mv/40 m/S** |
| 4 | 6/58 | 6/53 | 5/50 | 6/53 | ND | ND | ND | ND |
| 40 | 6.5/55.7 | 8/57.8 | 13.3/55 | 10.3/58.2 | ND | ND | ND | ND |
| 68 | 4.3/46.5 | 3.3/41.2 | 6.4/51.2 | 5.9/49.4 | ND | ND | ND | ND |
| 72 | ND | ND | ND | ND | 6.1/39.9 | 1.5/40.2 | 8.4/42.7 | 3.7/40.2 |
| 94 | 1.1/not evaluable | 1.2/50.0 | 3.3/61.0 | 5.8/50.0 | 2.2/37.1 | 0/0 | 7/43.9 | 2.4/43.6 |

**Table 1.** Evolution with time of sensory action potentials (µV) and compound motor action potentials (mV) and motor conduction velocities

(ms^-1^) recorded between the elbow and wrist for the upper limbs and the knee and ankle for the lower limbs. ‘Months’ correspond to the number

of months between the onset of symptoms as reported by the patient and the date of the electrophysiological evaluation. ND: not done. L: left. R: right. N: lower limit of normal.

|  |  | 4-18 months | | 40 months | | 68-72 months | | 74 months | |
| --- | --- | --- | --- | --- | --- | --- | --- | --- | --- |
|  |  | Right | Left | Right | Left | Right | Left | Right | Left |
| Upper limbs | Abductor policis brevis |  | SNC | SNC | CNC |  | CNC |  |  |
|  | First dorsal interosseus |  | N | SNC | CNC |  | CNC | CNC |  |
|  | Abductor digiti minimi |  | N | SNC | CNC |  |  |  |  |
|  | Extensor carpi radialis longus |  | N | SNC | SNC |  | CNC |  |  |
|  | Brachioradialis |  | N |  |  |  |  |  |  |
|  | Palmaris longus |  | N |  |  |  |  | SNC |  |
|  | Trapezius |  |  |  |  | SNC |  | SNC |  |
| Lower limbs | Vastus lateralis |  |  |  |  | SNC | ANC |  | CNC |
|  | Gastrocnemius medialis |  |  |  |  | SNC | SNC |  | ANC |
|  | Tibialis anterior |  |  |  |  | SNC | ANC | CNC |  |
|  | Extensor digitorum brevis |  |  |  |  | CNC |  |  |  |
|  | Biceps brachialis |  |  |  |  |  |  |  | CNC |
|  | Deltoidus |  |  |  |  |  |  | CNC |  |
| Other | Mentalis |  |  |  |  |  |  |  | N |

**Table 2.**

Evolution of electroneuromyography according to disease evolution. The results of needle examination are as follow: slight neurogenic changes (SNC): polyphasic motor unit potentials (MUPs) or MUPs with increased size; active neurogenic changes (ANC): fasciculation potentials, fibrillations potentials, or positive sharp waves; chronic neurogenic changes (CNC): loss of MUPs or high firing rate.

**Reference**

1. Cruccu, G, Aminoff, M J, Curio, G, Guerit, J M, Kakigi, R, Mauguiere, F, Rossini, P M, Treede, R D and Garcia-Larrea, L**: Recommendations for the clinical use of somatosensory-evoked potentials***. Clin Neurophysiol*. 2008**, 11**9:1705-19
